# Exploring the Potential Role of Quercetin Dihydrate Against Carbon Tetrachloride Induced Oxidative Stress in Mice: A Randomized Control Trial

**DOI:** 10.1002/fsn3.70793

**Published:** 2025-08-21

**Authors:** Farzeen Asghar, Imran Ullah Shah, Sajid Tahir, Abbas Khan, Zahoor Ahmed, Huriza Shakoor, Wayna Khalid, Uswah Saifullah, Komal Ajmal, Noreen Asghar, Mahboobullah Ahmadi

**Affiliations:** ^1^ Department of Nutrition and Health Promotion University of Home Economics Lahore Lahore Pakistan; ^2^ Department of Physiology University of Veterinary and Animal Sciences Lahore Pakistan; ^3^ School of Food and Nutrition Anhui Agricultural University Hefei China; ^4^ Human Nutrition and Dietetics School of Food and Agricultural Sciences, University of Management and Technology Lahore Pakistan; ^5^ Institute of Microbiology University of Veterinary and Animal Sciences Lahore Pakistan; ^6^ Department of pre‐Clinic, Faculty of Veterinary Science Afghanistan National Agricultural Sciences and Technology University Kandahar Afghanistan

**Keywords:** kidney, liver, mice ccl_4_ oxidative stress, muscles, Quercetin Dihydrate

## Abstract

This study investigated the protective efficacy of Quercetin Dihydrate against carbon tetrachloride‐(CCl_4_) induced oxidative stress using an animal model. A total of 24 healthy male mice were randomly divided into four groups. The first group served as control; the second group was treated with CCl_4_ (1 mL/kg b.w) intraperitoneally on day 21, and the remaining two treatment groups received varying daily doses of Quercetin Dihydrate (60, 120 mg/kg b.w) for 20 days and carbon tetrachloride (1 mL/kg b.w) intraperitoneally on day 21. We assayed biomarkers of oxidative stress, malondialdehyde (MDA), and catalase (CAT) in the liver, muscles, and kidneys. Moreover, carbon tetrachloride induces toxicity, which was associated with increased MDA levels (*p* ≤ 0.05) in the Control Positive group, indicating oxidative stress in mice. Quercetin Dihydrate supplementation decreased the MDA activity in the liver, muscle, and kidneys. The antioxidant activities of CAT were decreased (*p* ≤ 0.05) after carbon tetrachloride exposure. Quercetin Dihydrate administration normalized CCl_4_‐induced changes and significantly restored the redox status. In conclusion, by improving the oxidative stress markers, Quercetin Dihydrate exerted significant protection and antioxidant activities against carbon tetrachloride‐induced oxidative stress.

## Graphical abstract

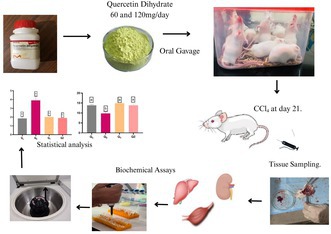

## 1 Introduction

Cellular oxygenation (oxidation) is a significant step in cellular metabolism, physiologically and pathologically associated with free radicals and reactive oxygen species formation (ROS) (Aramouni et al. 2023). Oxidative stress is an imbalance between reactive oxygen species and antioxidants in the body, disrupting redox control and alterations in proteins or DNA (Sies 1985). ROS are required for redox signaling and cell homeostasis in a controlled amount (Sies et al. 2017). However, there is an increase in the synthesis of ROS. In that case, there is a decrease in scavenging capability, resulting in the accumulation of free radicals and redox imbalance, ultimately leading to oxidative damage to the cell. Excessive production of free radicals can have detrimental effects on the body, leading to oxidative stress in the liver and kidney, which increases the risk of several diseases including cellular malfunction, cardiovascular diseases, cancer, diabetes, obesity, nephrotoxicity, and liver diseases, and raises public health concerns (Mishra et al. 2018; Safhi 2018). Nevertheless, the human body possesses defense mechanisms that might mitigate the effects of oxidative stress, including antioxidants (Misrani et al. 2021). The alteration of enzymatic antioxidants, including the enzyme superoxide dismutase (SOD), catalase (CAT), glutathione peroxidase (GPx), and NADPH‐quinone oxidoreductase‐1 (NQO1), contributes to cellular protection against oxidative stress (He et al. 2017).

Carbon tetrachloride (CCl_4_) is a colorless, organic solvent used for studying hepatotoxicity in experimental animals, as well as hepatic cirrhosis, fibrosis, liver damage, chemical hepatitis models, nephrotoxicity, and renal failure models (Hussain et al. 2017; Teschke 2018). It is highly lipophilic and can distribute throughout the lipid‐rich areas of the body (Srinivasan et al. 2007; Unsal et al. 2020). The process of toxicity occurs when CCl_4_ is converted into free radicals, specifically trichloromethyl and peroxy‐trichloromethyl radicals, through a biotransformation process that is initiated by cytochrome P450. These radicals then interact with proteins and Deoxyribonucleic Acid (DNA), leading to disruption in lipid metabolism (Dai et al. 2021). Furthermore, the metabolism of CCl_4_ triggers the activation of Kupffer cells by elevating intracellular calcium levels. This activation stimulates Kupffer cells to secrete pro‐inflammatory cytokines, which contribute to the development of oxidative stress (Pulli et al. 2015). Consequently, oxidative stress is induced by the depletion of reductants and the inhibition of antioxidant enzymes (Habashy et al. 2021). Many clinical studies have consistently shown that the application of CCl_4_ leads to a significant elevation of lipid peroxidation byproducts (Malondialdehyde, Lipid Peroxidation, Thiobarbituric Acid Reactive Substances), a marked rise in Deoxyribonucleic Acid (DNA) damage, and a higher level of protein oxidation products in the liver, tissues, and kidneys (Mazani et al. 2020). Scientists are focusing on finding plant‐based supplements and an antioxidant‐rich diet that can lower the excessive free radical production and suppress oxidative stress‐related complications (Al‐Qabba et al. 2020).

Quercetin is a natural flavonoid that belongs to polyphenols and is found in many vegetables and fruits. Quercetin Dihydrate are 2‐(3,4‐dihydroxyphenyl)‐3,5,7‐trihydroxy‐4H1‐benzopyran‐4‐one Dihydrate and 3,3′,4′,5,7‐pentahydroxyflavone Dihydrate. The chemical formula for Quercetin Dihydrate is C_15_H_10_O_7_•2H_2_O (C_15_H_14_O_9_) (Inger‐Lise Karin et al. 2024). Quercetin Dihydrate is a commercially available synthetic chemical molecule that exists as a bright yellow powder. The Dihydrate form of Quercetin, which is commercially available and more stable, has a triclinic crystal structure (Klitou et al. 2023). It has been researched experimentally using a variety of approaches and has been recognized as the most thermodynamically stable form under ambient conditions. Quercetin is available in anhydrous (QA), monohydrate (QMH), and Dihydrate (QDH) forms (Spiteri et al. 2018). Quercetin exhibits extremely low water solubility, leading to challenges in the crystallization of quercetin from aqueous solutions. Consequently, Dimethyl Sulfoxide‐water combinations were employed to enhance crystallization yield and obtain bigger crystals, as **Dimethyl Sulfoxide** (DMSO) may solubilize a broad spectrum of ordinarily insoluble or sparingly soluble compounds (Klitou et al. 2019).

Quercetin, a well‐known antioxidant, alleviates oxidative damage caused by acrylamide, radiation‐induced cerebral injury, cadmium fluoride‐induced neurological conditions associated with ROS and nerve damage in rats (Ola et al. 2016). The protective role of quercetin has also been reported in nephrotoxicity induced by gold nanoparticles (GNPs) and inflammatory kidney injury. It demonstrates significant alterations by reduction in glutathione levels and elevation in MDA activity (Abdelhalim et al. 2018). Quercetin can activate the AMPK‐p38 MAPK pathway, enhancing glucose uptake in skeletal muscle, which demonstrates its potential for the treatment of diabetes (Dhanya et al. 2017).

In a research conducted on cervical cancer cell line, it was found that Quercetin Dihydrate possesses anti‐cancer and anti‐proliferative properties, which may serve as alternative or adjunctive cancer therapies (Yadav et al. 2022).

This experimental design study aimed at exploring the antioxidant capacity of Quercetin Dihydrate using a mouse animal model to explore its potential health benefits against carbon tetrachloride‐induced toxicity on the redox state of mice by regulating oxidative stress‐related conditions. Furthermore, we measured oxidative stress markers, such as CAT and MDA, in liver, muscle, and kidney tissues.

## 2 Materials and Methods

### 2.1 Experimental Animal

20 healthy adult male mice aged 14–18 weeks and weighing 30–42 g were collected from a local supplier and housed in the animal house of the Department of Physiology at the University of Veterinary and Animal Sciences in Lahore. All mice were kept in a temperature‐regulated environment maintained at 25°C. Mice were permitted to adjust to the new surroundings for 1 week. 20 mice were divided into four groups, with each comprising five mice. In the animal trial, plastic cages were designed to house the animals, utilizing wood litter bedding that was changed weekly to ensure hygiene and prevent disease. Additionally, the temperature within the cages was regulated to maintain a consistent 24°C ± 2°C. All mice were weighed using an electronic balance prior to the commencement of treatment. The general features and physical condition of the mice were carefully assessed daily during the trial period. Quercetin Dihydrate (> 98% HPLC) powder was purchased from Sigma‐Aldrich, Merck. A certificate of ethical procedures was acquired from ORIC, UHE Lahore, before the commencement of the experiment.

**Animals were divided into four groups**.

## 3 Q_C_ (Control Negative)

Mice in this group were administered standard diet and water, subsequently receiving a single dose of normal saline intraperitoneally (I/P) at day 21. The composition of standard laboratory chow diet contains: crude protein = 19%, crude fat = 3.3%, crude fiber = 4.9%, crude ash = 6.4%, starch = 36.5%, sugar/dextrins = 4.7%, carbohydrate = 58%, protein = 33%, and fat = 9%. In order to equalize the stress of the gavage process compared to other groups, they received tap water by administering oral gavage daily till 21 days of trials (Molavi Vasei et al. 2024; Ricci 2015).

## 4 Q_0_ (Control Positive)

Mice were provided with a standard diet and water. The mice in that group were administered normal saline orally for 20 days, after which they received an intraperitoneal injection of a 1:1 ratio of CCl_4_ and olive oil (1 mL/kg body weight) on day 21 (Algefare et al. 2024; Al‐Seeni et al. 2016).

1. **Q**_**1**_ **(60mg Quercitin Dihydrate)**
  Mice in this group were supplemented with Quercetin Dihydrate (60 mg/day) orally with diet, followed by administration of CCl_4_ + Olive oil; 1:1 (1 mL/kg b.w) at day 21 (Algefare et al. 2024; Al‐Seeni et al. 2016).
2. **Q**_**2**_ **(120mg Quercitin Dihydrate)**
  Mice in this group were supplemented with Quercetin Dihydrate (120 mg/day) orally for 20 days, along with diet, after administration of CCl_4_ + Olive oil; 1:1 (1 mL/kg b.w) on day 21.

### 4.1 Sampling and Storage

At the end of the 21‐day period, the mice were fasted for about 12 h. Then, the mice were anesthetized with light ether anesthesia. Blood was obtained through cardiac puncture. Following the collection of blood, the mice were decapitated to get muscle tissue. The abdomen was opened, and both the liver and kidneys were collected. Tissue samples were collected, labeled, and stored at −20°C in preparation for biochemical analysis.

### 4.2 Biochemical Assays

#### 4.2.1 Tissue Homogenization

To assess the activities of MDA and CAT, liver, muscle, and kidney slices were homogenized in a homogenization buffer. The liver, kidney, and muscle tissues were individually weighed, ground, and homogenized in ice‐cold 0.01 M phosphate‐buffered saline (pH 7.4) using a homogenizer. The homogenates were centrifuged at 5000 × g at 4°C for 20 min. The resulting supernatant was utilized for the biochemical assessment of MDA and CAT levels (Shehzad et al. 2024).

### 4.3 Assessment of Oxidative Stress Marker

Malondialdehyde (MDA) results from the lipid peroxidation of polyunsaturated fatty acids. The end product of lipid peroxidation, MDA, interacts with thiobarbituric acid (TBA), resulting in a red‐colored compound at a pH of 3.5. The concentration of this red‐colored product corresponds proportionally to the absorbance of MDA (Ohkawa et al. 1979). The absorbance was measured at a wavelength of 535 nm, and the concentration of MDA in tissue was determined using tetraethoxypropane as an external standard. The results are presented as μmol MDA/L tissue:

y=71.562×–3.336

### 4.4 Determination of Antioxidant Activities

The CAT activity assay protocol was described. The undecomposed hydrogen peroxide (H_2_O_2_) interacts with ammonium molybdate ((NH_4_)2MoO_4_), resulting in the appearance of a yellow color. The peak absorption value occurs at 374 nm. This technique involves the incorporation of a correction factor to mitigate interferences caused by the presence of proteins and amino acids in analytes (Hadwan and Abed 2016).

$$\mathrm{CAT}\;\text{activity test}=2.303÷t\times \left(\log\;\mathrm{So}÷S-M\right)\times V_{t}÷\mathrm{Vs}$$

### 4.5 Statistical Analysis

Data obtained from biochemical tests were analyzed using the Statistical Analysis Software for the Social Sciences (SPSS), version 25.0 (IBM Corp., Armonk, N.Y., USA) and represented as mean ± SEM. The difference among means was checked using a one‐way ANOVA followed by post hoc and Turkey. A value of *p* < 0.05 was considered an indicator of statistically significant. GraphPad Prism was used to represent the data graphically.

## 5 Results

### 5.1 Impact of Quercetin Dihydrate on Liver Redox Status Against Carbon Tetrachloride (CCl_4_ ) Induced Oxidative Stress in Mice

This study examines the effect of Quercitin Dihydrate on the redox status of the liver in mice that have been exposed to oxidative stress induced by CCl_4_. Q_C_ (control negative), Q_0_ (Control positive + CCl_4_ exposure), Q_1_ (60mg Quercitin Dihydrate + CCl_4_), and Q_2_ (120 mg Quercitin Dihydrate + CCl_4_) were examined (Table 1).

**TABLE 1:** Effects of Quercitin Dihydrate on redox status against carbon tetrachloride (CCl_4_) induced oxidative stress in liver, muscle, and kidney of experimental groups.

Parameters		Q_C_	Q_0_	Q_1_	Q_2_	*p*‐value
Liver	MDA	1.85 ± 0.12^b^	3.91 ± 0.45^a^	2.01 ± 0.01^b^	1.89 ± 0.028^b^	0.001
	CAT	13.87 ± 0.43^a^	9.79 ± 0.88^b^	14.87 ± 0.04801^a^	13.83 ± 0.96^a^	0.001
Muscle	MDA	2.07 ± 0.057^b^	4.22 ± 0.24^a^	1.97 ± 0.066^b^	1.71 ± 0.024^b^	0.001
	CAT	12.55 ± 0.22^a^	9.72 ± 0.15^b^	12.69 ± 0.099^a^	13.58 ± 1.04^a^	0.003
Kidney	MDA	2.00 ± 0.074^b^	3.66 ± 0.28^a^	1.88 ± 0.03^b^	1.99 ± 0.14^b^	0.001
	CAT	12.18 ± 1.36	10.59 ± 0.71	12.01 ± 0.18	12.67 ± 0.37	0.333

*Note:* Mean values are expressed + standard error (SE) for five mice per group. Letters (a, b) in the superscript represent mean differences for each instar group analyzed using one‐way ANOVA (*p* < 0.05). Q_C_ = Control Negative (diet and water subsequently receiving a single dose of normal saline intraperitoneally (I/P) at day 21); Q_0_ = Control Positive (administered normal saline orally for 20 days, after which they received an intraperitoneal injection of a 1:1 ratio of CCl_4_ and olive oil (1 mL/kg body weight) on day 21); Q_1_ = supplemented Quercetin Dihydrate (60 mg/day) orally with diet followed by administration of CCl_4_ + olive oil; 1:1 (1 mL/kg b.w) at day 21; Q_2_ = Quercetin Dihydrate (120 mg/day) orally for 20 days along with diet after administration of CCl_4_ + olive oil; 1:1 (1 mL/kg b.w) at day 21.

**FIGURE 1:**
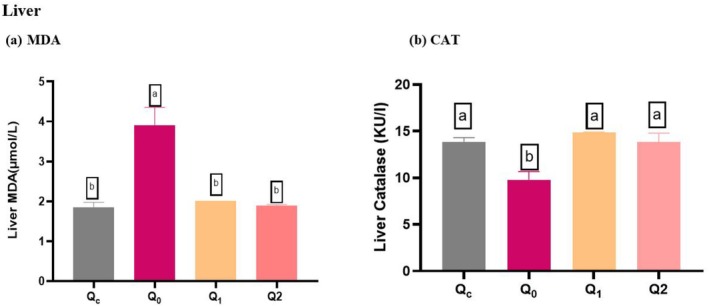
Oxidative stress in Liver MDA levels and CAT enzymatic activities. (a) MDA levels; Results are presented as mean ± SD (*n* = 4 in each group). *Significant difference observed at p Superscripts a*‐ values referred to control positive (means high oxidative stress); and b*‐ values referred to lower oxidative stress (low MDA level). (b) CAT levels; Superscripts b*‐ values referred to control positive (means high oxidative stress); and a*‐ values referred to low oxidative stress (high CAT level). Different superscript^−b^ on bars mean that the difference between groups is statistically significant (*p* < 0.05). Q_C_ = Control Negative (diet and water subsequently receiving a single dose of normal saline intraperitoneally (I/P) at day 21), Q_0_ = control Positive (administered normal saline orally for 20 days, after which they received an intraperitoneal injection of a 1:1 ratio of CCl_4_ and olive oil (1 mL/kg body weight) on day 21), Q_1_ = supplemented Quercetin Dihydrate (60 mg/day) orally with diet followed by administration of CCl_4_ + Olive oil; 1:1 (1 mL/kg b.w) at day 21, Q_2_ = Quercetin Dihydrate (120 mg/day) orally for 20 days along with diet after administration of CCl_4_ + Olive oil; 1:1 (1 mL/kg b.w) at day 21.

The level of MDA, which is a biomarker for lipid peroxidation, was found to be considerably increased in Q_0_ (Control Positive) compared to the Qc (Control Negative). The pretreatment with Quercitin Dihydrate resulted in a decrease (*p* < 0.005) in the liver MDA level as compared to the control positive group (Figure 1).

The oxidative stress induced mice showed reduced CAT level in Q_0_ (Control Positive). The liver CAT activity was significantly lower in control positive compared to control negative, whereas Quercitin Dihydrate significantly increased (*p* < 0.05) in the liver CAT activity in mice (Table 1). However, there is no significant difference between Quercitin Dihydrate‐treated groups and the control negative group, which means that Quercitin Dihydrate reduces the oxidative stress in the liver, similar to that in the control negative (Unexposed Group) (Figure 1).

### 5.2 The Potential of Different Doses of Quercetin Dihydrate on Oxidative Stress Level in Muscles

**FIGURE 2:**
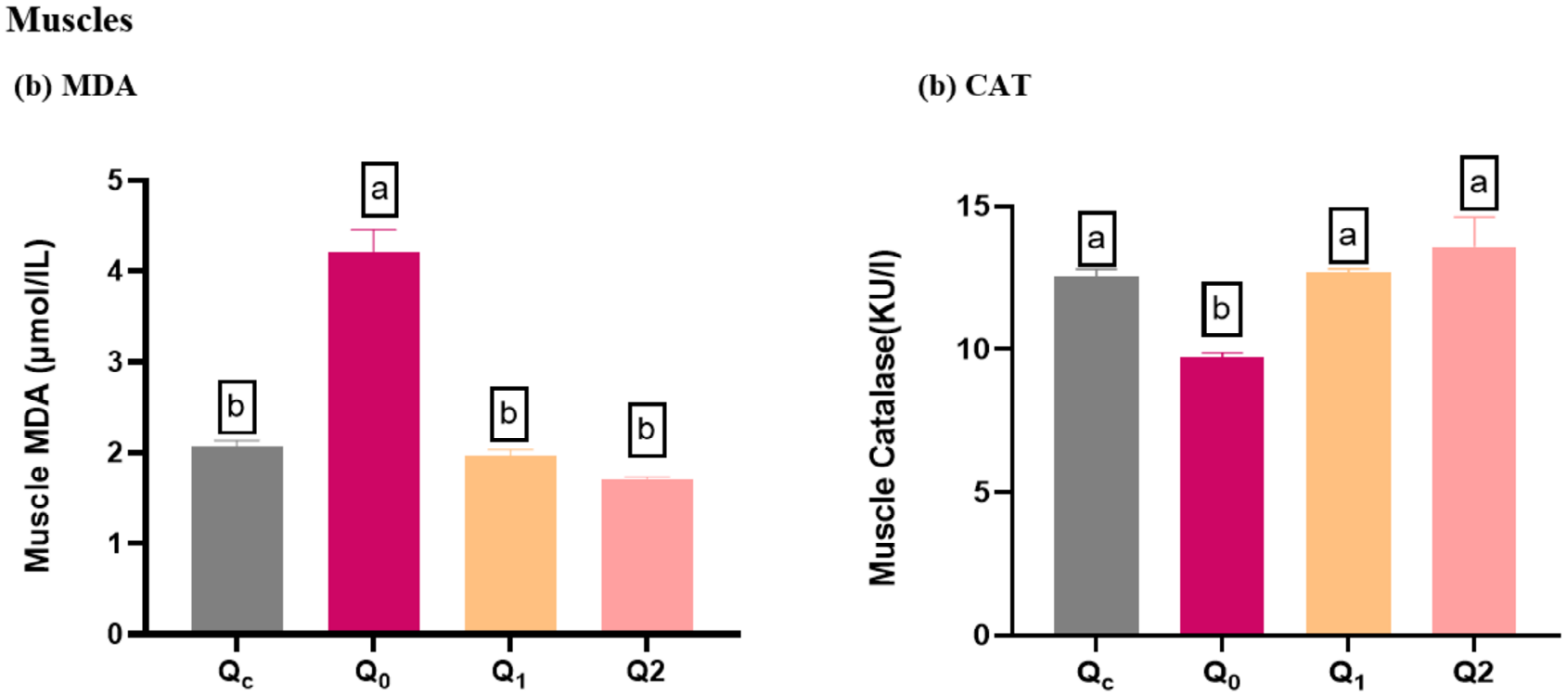
Oxidative stress in Muscles MDA levels, and CAT enzymatic activities (a) MDA levels; Results are presented as mean ± SD (*n* = 4 in each group). *Significant difference observed at p Superscripts a*‐ values referred to control positive (means high oxidative stress); and b*‐ values referred to lower oxidative stress (low MDA level). (b) CAT; Superscripts b*‐ values referred to control positive (means high oxidative stress); and a*‐ values referred to low oxidative stress (high CAT level). Different superscript^−b^ on bars mean that the difference between groups is statistically significant (*p* < 0.05). Q_C_ = Control Negative (diet and water subsequently receiving a single dose of normal saline intraperitoneally (I/P) at day 21), Q_0_ = Control Positive (administered normal saline orally for 20 days, after which they received an intraperitoneal injection of a 1:1 ratio of CCl_4_ and olive oil (1 mL/kg body weight) on day 21), Q_1_ = supplemented Quercetin Dihydrate (60 mg/day) orally with diet followed by administration of CCl_4_ + Olive oil; 1:1 (1 mL/kg b.w) at day 21, Q_2_ = Quercetin Dihydrate (120 mg/day) orally for 20 days along with diet after administration of CCl_4_ + Olive oil; 1:1 (1 mL/kg b.w) at day 21.

The findings of the present study demonstrated that the MDA level in muscle was significantly increased in mice of Q_0_ (Control Positive) compared to Qc (Control Negative). Specifically, Q_0_ (Control Positive) exhibited an MDA level of, indicating increased oxidative stress. However, Quercetin Dihydrate significantly decreased (*p* < 0.001) MDA level compared to Q_0_ (Control Positive) in the liver (Figure 2).

The CAT level in muscle tissues was decreased in Q_0_ (Control Positive) compared to Qc (Control Negative). However, Quercetin Dihydrate has significantly increased (*p* < 0.001) Muscle CAT activity compared with the Control Positive (Table 1).

### 5.3 Effect of Quercetin Dihydrate Against Oxidative Stress by CCl_4_ in the Kidney

**FIGURE 3:**
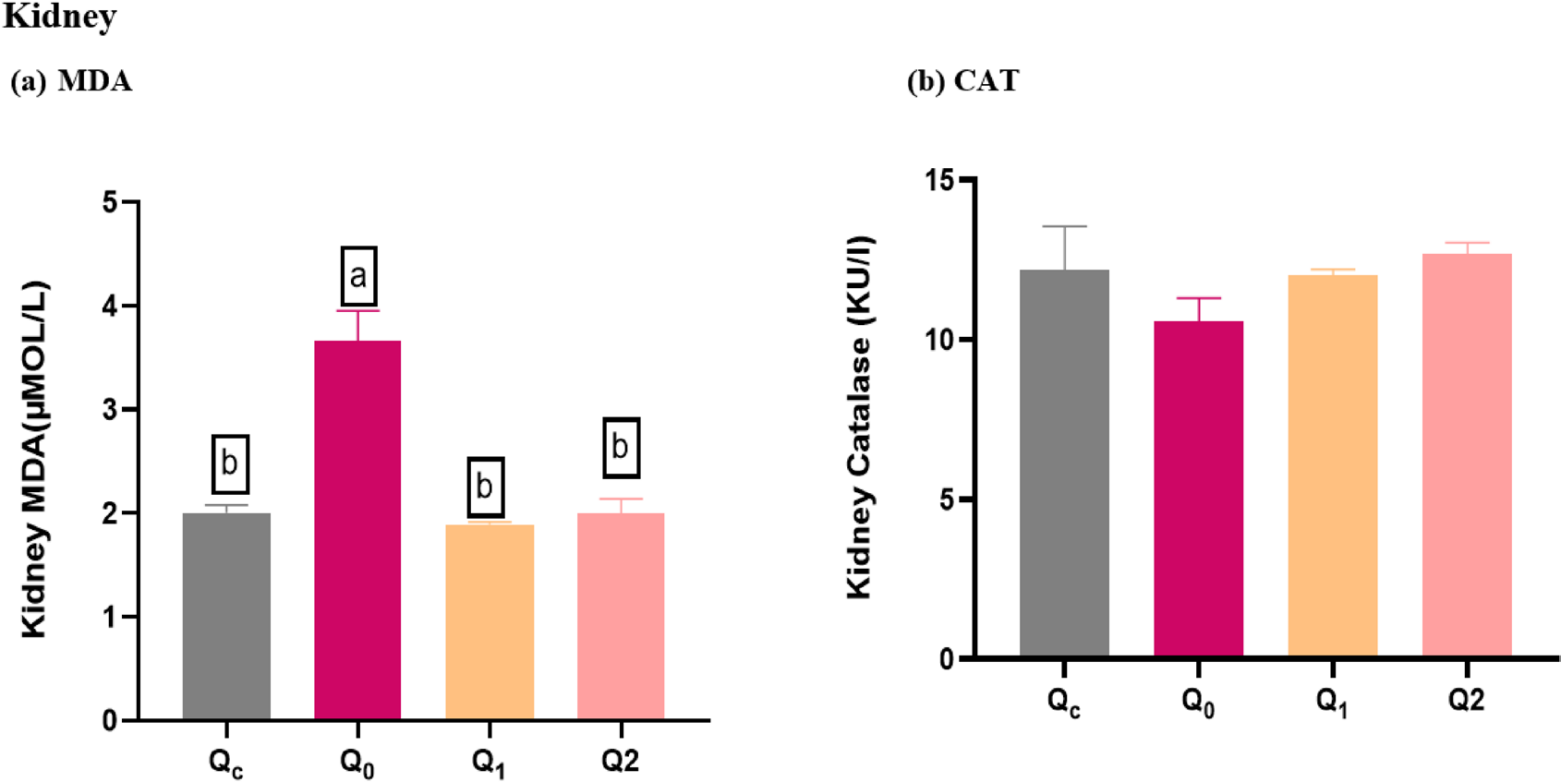
Oxidative stress in kidney MDA levels and CAT enzymatic activities. (a) MDA levels; Results are presented as mean ± SD (*n* = 4 in each group). *Significant difference observed at p. Superscripts a*‐ values referred to control positive (means high oxidative stress); and b*‐ values referred to lower oxidative stress (low MDA level). (b) CAT levels; Superscripts b*‐ values referred to control positive (means high oxidative stress); and a*‐ values referred to low oxidative stress (high CAT level). Different superscript^−b^ on bars means that the difference between groups is statistically significant (*p* < 0.05). Q_C_ = control negative (diet and water subsequently receiving a single dose of normal saline intraperitoneally (I/P) at day 21), Q_0_ = control positive (administered normal saline orally for 20 days, after which they received an intraperitoneal injection of a 1:1 ratio of CCl_4_ and olive oil (1 mL/kg body weight) on day 21), Q_1_ = supplemented Quercetin Dihydrate (60 mg/day) orally with diet followed by administration of CCl_4_ + Olive oil; 1:1 (1 mL/kg b.w) at day 21, Q_2_ = Quercetin Dihydrate (120 mg/day) orally for 20 days along with diet after administration of CCl_4_ + Olive oil; 1:1 (1 mL/kg b.w) at day 21.

The kidney MDA level was significantly increased in Q_0_ (Control Positive) compared to the Q_C_ (Control Negative), indicating elevated oxidative stress in the kidneys of the carbon tetrachloride CCl_4_ exposed group of mice. The Q_0_ (Control Positive) showed elevated MDA levels compared to the Q_C_ (Control Negative). However, the treatment groups supplemented with Quercetin Dihydrate (Q_1_ and Q_2_) have significantly decreased (*p* = 0.01) the kidney MDA level compared to Q_0_ (Control Positive) (Figure 3).

In terms of antioxidant enzymes, there is no significant change (*p* = 0.333) in the CAT level of kidney tissues with Quercetin Dihydrate supplementation in mice compared with the control Positive groups. This indicates that Quercetin Dihydrate did not enhance the antioxidant defense mechanisms in kidney tissue under CCl_4_‐induced oxidative stress (Table 1).

## 6 Discussion

The primary objective of the present study was to explicate the antioxidant potential of Quercetin Dihydrate in an experimental mice model in which oxidative stress of the liver, muscle, and kidney was induced by CCl_4_. Additionally, this research particularly aimed to evaluate the potential therapeutic advantages of various doses of Quercetin Dihydrate to assess oxidative stress biomarkers in different organs.

The researchers demonstrated that intraperitoneal administration of 1.25 mL/kg body weight of CCl_4_ in liquid paraffin (1:1) eventually resulted in elevated MDA levels in rats. The elevated levels of MDA are a recognized indicator of carbon tetrachloride‐induced lipid peroxidation, leading to kidney and liver injury (Al‐Yahya et al. 2013). The results of this research indicated significant differences in some of the measured biochemical parameters linked to oxidative stress, which increased in the liver, muscle, and kidney of CCl_4_‐induced rats compared to the control group. These parameters were subsequently reversed in the groups treated with Quercetin Dihydrate.

At a physiological level, as well as pathological, ROS are essential for sustaining various critical biological processes, maintaining redox balance, and regulating significant transcription factors (Liguori et al. 2018). Conversely, an overproduction of ROS disrupts redox balance, resulting in oxidative stress, subsequently disrupting essential biomolecules such as proteins, DNA, and membranes, as well as reduced antioxidant levels in tissues (Alshammari et al. 2017).

To mitigate this problem, all organisms exhibit specialized enzyme defense systems, indicating various activities in response to ROS (Kozlov et al. 2024). Antioxidants are compounds that may affect the oxidant–antioxidant balance in the body by neutralizing pro‐oxidant molecules. Quercetin is a flavonol, an essential class of secondary plant phenolic metabolites that are natural antioxidants that regulate cellular redox balance (Zaplatic et al. 2019). The antioxidant activity of Quercetin has been documented due to its ability to scavenge ROS (Batiha et al. 2020). It is capable of scavenging a variety of free radicals, including hydrogen peroxide (H_2_O_2_), nitric oxide (NO), and hydroxyl radical (OH‐) (Saikia 2022). Quercetin Dihydrate, a commercially available variant of this compound, has undergone extensive experimental investigation employing a diverse array of techniques and has been recognized as the most thermodynamically stable form under ambient conditions (Klitou et al. 2020).

Our study revealed that a single dosage of CCl_4_ administration resulted in oxidative stress in the liver, muscle, and kidney as evidenced by increased concentration of MDA. The findings of our study align with previous research (Saoudi et al. 2021), indicating that the administration of CCl_4_ (2 mg/kg) increases MDA levels in the kidney and liver, concurrently reducing the enzyme activity of antioxidant enzymes (El‐haskoury et al. 2018).

MDA is a lipid peroxidation byproduct that typically increases with oxidative stress. Lipid peroxidation is a significant organic manifestation of oxidative stress caused by the reactivity of oxygen‐free radicals. Considerable attempts have been made to provide effective analytical procedures for assessing the level of lipid peroxidation in tissue homogenates, organs, or whole organisms (Senthilkumar et al. 2020). The liver is essential for metabolism, storage, secretion, and the detoxification of toxic substances. Oxidative stress from exposure to numerous xenobiotics, including free radicals and hydrogen peroxides, can adversely impact liver function (Alkinani et al. 2021). The present findings showed that MDA levels were increased in response to carbon tetrachloride treatment. However, in the liver, the MDA level in mice administered with Quercetin Dihydrate (120 mg/kg/b.w) showed the lowest MDA level compared to those administered Quercetin Dihydrate (60 mg/kg/b.w). However, both groups show the same superscript (b), indicating that the differences in MDA levels are not statistically significant. Our findings are supported by previous research, which showed that Quercetin (50 mg/kg/b.w) manifests notable hepatoprotective properties and improves liver function by preventing the elevation in liver lipid peroxidation and reducing antioxidant enzyme activities (Nassef et al. 2020; Nouri et al. 2019).

Skeletal muscles constitute approximately 40% of total body weight, facilitating locomotion and maintaining posture through contraction and relaxation mechanisms (Mesinovic et al. 2019). Upon exposure to CCl_4_, there is an elevation in the generation of ROS. Reports indicate that CCl_4_ toxicity induces oxidative stress and enhances the production of ROS, which are the primary mechanisms that cause its toxic effects. This leads to cellular structural damage and muscle impairment, ultimately contributing to muscular loss, neuromuscular degeneration, and fiber depletion (Scicchitano et al. 2018).

In this study, the administration of CCl_4_ significantly increased oxidative stress in muscle tissue and markedly elevated the concentration of MDA, which is the end product of lipid peroxidation. The highest level for MDA was seen in the positive control (Q_0_), which was greater than the treatment (Q_2_). It was evident from the current results that treatment with Quercetin Dihydrate (Q_1_ = 60 mg, Q_2_ = 120 mg Quercetin Dihydrate) resulted in a significant reduction in MDA production, thereby inhibiting oxidative stress when compared to the positive control (Q_0_). In addition, a previous study demonstrated similar results regarding Quercetin/β‐cyclodextrin transdermal gel's effects on oxidative stress indicators following skeletal muscle damage. In this investigation, 98 h post‐experimental muscle damage, the subjects demonstrated elevated Thiobarbituric Acid Reactive Substances TBARS levels and antioxidant enzyme activity. The Quercetin/β‐cyclodextrin transdermal gel diminishes oxidative stress indicators during skeletal muscle damage, regardless of phonophoresis application (Sousa Filho et al. 2021). The kidney is a principal organ within the human body, keeping critical functions in the regulation of blood pressure, the maintenance of hemostasis, and the facilitation of detoxification and waste excretion (Ali et al. 2021). It serves as the primary pathway for the elimination of numerous xenobiotics, drugs, and their metabolites (Asejeje et al. 2020).

The present findings of renal toxicity were determined in animals by the administration of Quercetin Dihydrate to 20 mice. We observed that the level of MDA in the kidney tissues was significantly elevated by CCl_4_ exposure. Pretreatment with Quercetin Dihydrate (Q_1_ = 60 mg, Q_2_ = 120 mg) reduced oxidative stress by significantly down regulating the levels of MDA in the kidney. This MDA‐lowering effect could be attributed to the Quercetin Dihydrate, which showed an ameliorative effect on oxidative stress and reduced CCl_4_‐induced toxicity in kidneys. Multiple studies have indicated that decreasing MDA levels in the kidneys could be one of the possible ways of lowering oxidative stress, which was also supported by earlier studies. The results confirm previous similar research, which also indicated that supplementation with quercetin (50 mg/kg/b.w) for a consecutive 28 days mitigates the fipron‐induced nephrotoxicity by decreasing MDA level in the kidney (Uzunhisarcikli et al. 2023). Additionally, another research study had shown that Quercetin supplementation (50‐100 mg/kg/day) for 4 weeks significantly attenuated CuSO_4_‐induced nephrotoxicity, reducing the MDA levels (Peng et al. 2021).

Excessive production of ROS can contribute to the oxidative degradation of molecules that contribute to diabetes and several ailments, including cancer, neurological diseases, and cardiovascular diseases (Gyurászová et al. 2020). Redox reactions play a crucial role in cellular homeostasis, which depends on the interplay between oxidants and the defense system, which comprises reductants and antioxidant enzymes. The organisms have a defense mechanism that removes the free radicals by the exogenous and endogenous antioxidant systems (Moussa et al. 2019). CAT is a significant antioxidant enzyme within the cellular systems of organisms. CAT is essential in cellular defense by reducing oxidative stress from ROS. It demonstrates one of the most excellent catalytic efficiencies among enzymes, with a single CAT molecule turning millions of hydrogen peroxide (H_2_O_2_) molecules into water and oxygen per second (Anwar et al. 2024).

Data obtained from this investigation confirmed that CCl_4_ induced substantial oxidative stress in mice, as demonstrated by altered liver, muscle, and kidney antioxidant enzyme activity, with reduced CAT activity. Studies revealed that CAT activity was significantly decreased in the liver tissue of male mice exposed to CCl_4_ (1 mL/kg/b.w) toxicity (Thanebal et al. 2021). Multiple studies indicate that exposure to CCl_4_ in male rats results in decreased CAT activity due to disruption of the antioxidant defense system in the liver and kidneys (Cinar et al. 2024).

The liver is a vital organ involved in more than 500 metabolic processes within the biological system. Its primary function is the detoxification of poisons and harmful substances; however, it can also sustain damage from toxicants, which may disrupt its metabolic activities and result in acute liver failure (Ojeaburu and Oriakhi 2021). Our study indicated that the CAT activity in the liver was significantly decreased in CCl_4_ alone–treated mice. However, interestingly, our results also suggested that the supplementation of Quercetin Dihydrate (60 and 120 mg/kg) increases the CAT activity in the liver. As evidenced by the present study, Quercetin Dihydrate appears to possess the ability to decrease oxidative stress in pathological conditions by boosting the activity of antioxidant enzymes and increasing levels of CAT.

Our study is in line with other studies, which established that quercetin enhances CAT activity and decreases oxidative damage induced by CCl_4_ in the hepatic tissue of rats (Pingili et al. 2019). Quercetin exhibits its antioxidant action via many molecular mechanisms, including a direct method to neutralize excess ROS resulting from oxidative damage (Al‐Zharani et al. 2023).

Oxidative stress is a contributing factor through the production of ROS in the pathogenesis of chronic diseases linked to muscle loss (Scicchitano et al. 2018). Consequently, the reduction in enzyme activity leads to several adverse effects due to the accumulation of hydrogen peroxide and superoxide radicals. These observations also suggest that CAT activity in the muscles of male Wistar rats is reduced due to exposure to (1 mL/kg) CCl_4_ (Sokolović et al. 2018). A relatively limited number of research studies have explored the impact of Quercetin Dihydrate on the CAT levels in muscles.

The findings of this study demonstrated that decreased CAT activity was identified in the Q_0_ (Control Positive) group following CCl_4_ induction, in contrast to the Control Negative group in the Muscles. The data indicated that an increase in the activity level of this enzyme was observed in the Quercetin Dihydrate (60, 120 mg/kg) supplemented groups, compared to the control positive group values. This study demonstrates increased CAT enzyme activities in the various doses of Quercetin Dihydrate groups compared to the CCl_4_ group, which may be attributed to the observed antioxidant properties (Alm‐Eldeen et al. 2020).

Oxidative damage has significant effects, frequently seen by enhanced activity of oxygen detoxifying enzymes in the kidney (Mazher et al. 2021). In this study, the intraperitoneal injection of CCl_4_ (1 mL/kg/b.w) induced oxidative stress in Q_0_ (Control Positive) and decreased CAT activity in the kidney. These results are in accordance with the previous report suggesting that CCl_4_ increases oxidative stress and decreases antioxidant agents, including CAT, in the kidney tissues (El‐Haskoury et al. 2021). CAT activity is a commonly used test that can provide information about the cell's antioxidant defense system. On the other hand, in our research, the mice supplement CAT level was significantly decreased in the kidneys. However, CAT activity demonstrated trends toward significance (*p* = 0.33) in the kidney. In our body, the first line of defense is the GGG cytoplasm of the proximal tubules situated in the juxtamedullary cortex. At the same time, its expression is comparatively diminished in the proximal tubules of the superficial cortex. Conversely, CAT is absent in the glomeruli, distal tubules, loop of Henle, and collecting ducts (Hong and Park 2021).

## 7 Conclusion

In conclusion, the present study provides novel evidence that Quercetin Dihydrate significantly mitigates CCl_4_‐induced oxidative stress. Furthermore, oral administration of Quercetin Dihydrate at doses of 60 and 120 mg/kg body weight effectively protected mice tissues, including the liver, muscle, and kidney, from CCl_4_‐induced oxidative stress following 21 days of treatment. Quercetin Dihydrate improved antioxidant status by reducing lipid peroxidation, as evidenced by decreased MDA levels, and enhancing the activity of key antioxidant enzymes such as CAT. The results are consistent with previous studies that indicate blueberry polyphenols prevent liver fibrosis, control inflammation, and combat oxidative stress by modifying fibrotic markers, pro‐inflammatory cytokines, and increasing antioxidant activity. These effects suggest that Quercetin Dihydrate effectively neutralizes oxidative damage by CCl_4_ and significantly improves redox status in affected organs. Research findings are consistent with previous studies that indicate that Quercetin Dihydrate reduces liver fibrosis and atherosclerosis, controls inflammation, and combats oxidative stress by modifying fibrotic markers and pro‐inflammatory cytokines and increasing antioxidant activity. Currently, the study demonstrates the significant antioxidant potential of Quercetin Dihydrate. The detailed mechanism underlying the antioxidant potential of Quercetin Dihydrate can be further explored by studying the molecular pathways involved, which we were not able to perform this time, and can be added in our future studies. However, further research is required to elucidate the adverse effects of CCl_4_, including the analysis of gene expression indicators and metabolomics, in order to validate further the effects of Quercetin Dihydrate in prophylaxis and prevention of cell injuries.

## Author Contributions

**Farzeen Asghar:** conceptualization (equal), data curation (equal), formal analysis (equal), software (equal), writing – original draft (equal). **Imran Ullah Shah:** data curation (equal), resources (equal), software (equal), supervision (equal), validation (equal), writing – review and editing (equal). **Sajid Tahir:** formal analysis (equal), resources (equal), writing – review and editing (equal). **Abbas Khan:** data curation (equal), formal analysis (equal), software (equal), writing – review and editing (equal). **Zahoor Ahmed:** formal analysis (equal), software (equal), writing – review and editing (equal). **Huriza Shakoor:** formal analysis (equal), software (equal). **Wayna Khalid:** formal analysis (equal), software (equal), writing – review and editing (equal). **Uswah Saifullah:** formal analysis (equal), software (equal). **Komal Ajmal:** software (equal), visualization (equal). **Noreen Asghar:** software (equal), visualization (equal). **Mahboobullah Ahmadi:** investigation (equal), software (equal), writing – review and editing (equal).

## Conflicts of Interest

The authors declare no conflicts of interest.

## Data Availability

The data will be provided according to the requirments.
